# De Novo Lipogenesis and Cholesterol Synthesis in Humans with Long-Standing Type 1 Diabetes Are Comparable to Non-Diabetic Individuals

**DOI:** 10.1371/journal.pone.0082530

**Published:** 2013-12-23

**Authors:** Jennifer E. Lambert, Edmond A. Ryan, Alan B. R. Thomson, Michael T. Clandinin

**Affiliations:** 1 Alberta Institute for Human Nutrition, University of Alberta, Edmonton, Alberta, Canada; 2 Department of Medicine, University of Alberta, Edmonton, Alberta, Canada; University of Michigan Medical School, United States of America

## Abstract

**Background:**

Synthesis of lipid species, including fatty acids (FA) and cholesterol, can contribute to pathological disease. The purpose of this study was to investigate FA and cholesterol synthesis in individuals with type 1 diabetes, a group at elevated risk for vascular disease, using stable isotope analysis.

**Methods:**

Individuals with type 1 diabetes (n = 9) and age-, sex-, and BMI-matched non-diabetic subjects (n = 9) were recruited. On testing day, meals were provided to standardize food intake and elicit typical feeding responses. Blood samples were analyzed at fasting (0 and 24 h) and postprandial (2, 4, 6, and 8 hours after breakfast) time points. FA was isolated from VLDL to estimate hepatic FA synthesis, whereas free cholesterol (FC) and cholesteryl ester (CE) was isolated from plasma and VLDL to estimate whole-body and hepatic cholesterol synthesis, respectively. Lipid synthesis was measured using deuterium incorporation and isotope ratio mass spectrometry.

**Results:**

Fasting total hepatic lipogenesis (3.91±0.90% vs. 5.30±1.22%; P = 0.41) was not significantly different between diabetic and control groups, respectively, nor was synthesis of myristic (28.60±4.90% vs. 26.66±4.57%; P = 0.76), palmitic (12.52±2.75% vs. 13.71±2.64%; P = 0.65), palmitoleic (3.86±0.91% vs. 4.80±1.22%; P = 0.65), stearic (5.55±1.04% vs. 6.96±0.97%; P = 0.29), and oleic acid (1.45±0.28% vs. 2.10±0.51%; P = 0.21). Postprandial lipogenesis was also not different between groups (P = 0.38). Similarly, fasting synthesis of whole-body FC (8.2±1.3% vs. 7.3±0.8%/day; P = 0.88) and CE (1.9±0.4% vs. 2.0±0.3%/day; P = 0.96) and hepatic FC (8.2±2.0% vs. 8.1±0.8%/day; P = 0.72) was not significantly different between diabetic and control subjects.

**Conclusions:**

Despite long-standing disease, lipogenesis and cholesterol synthesis was not different in individuals with type 1 diabetes compared to healthy non-diabetic humans.

## Introduction

Individuals with type 1 diabetes are at a markedly higher risk of cardiovascular disease (CVD) mortality, despite lack of traditional risk factors [1]. Abnormalities in lipid and lipoprotein composition or metabolism may contribute to CVD risk, and may arise due to alterations in synthesis, absorption, or clearance.

De novo lipogenesis (DNL), the synthesis of fatty acids, is assumed to be a minor contributor to plasma triglyceride (TG) levels in healthy humans, but this may not be true for all disease states. DNL is elevated in states of glucose interolance such as type 2 diabetes [2] and non-alcoholic fatty liver disease (NAFLD) [3]. In these individuals, DNL has been reported to contribute significantly to both circulating TG levels and is highly associated with intrahepatic TG accumulation [3], [4]. Recent reports have indicated that up to half of individuals with type 1 diabetes have some degree of liver fat accumulation, which is associated with greater prevalence of vascular disease despite normal plasma cholesterol and TG levels [5]. NAFLD development is highly associated with insulin resistance [6], which may be more prominent in type 1 diabetic individuals than once appreciated [7], [8]. Animal models of type 1 diabetes suggest reduced DNL [9]–[11], but, to date, there have been no investigations of DNL in humans with type 1 diabetes. Further, the main products of lipogenesis are saturated fatty acids (FA), which may negatively influence cellular function and metabolism [12] and therefore contribute to the metabolic dysregulation observed in type 1 diabetes. The deuterium incorporation method is particularly suited to the measurement of DNL because is safe, can be used with a short measurement period (≤24 hours), rapidly equilibrates across body pools, and provides a highly sensitive and precise measurement of in vivo synthesis [13]. Typically when DNL is assessed, only the major product of DNL is measured (i.e. palmitate); however, using gas chromatography isotope-ratio mass spectrometry (GC-IRMS) allows for the measurement of the synthesis rate of individual fatty acids. For example, Bergman et al (2013) recently observed that individuals with type 1 diabetes had a lower amount of palmitoleic acid in plasma-TG compared to non-diabetic individuals, which was inversely related to adipocyte insulin sensitivity; these authors questioned whether this reduced content of 16∶1 was due to reduced dietary intake or hepatic synthesis and desaturation [14]. Therefore DNL may have quantitative and qualitative implications, and determination of its potential role in the increased vascular disease risk in type 1 diabetes is of importance.

As stated previously, the paradox in type 1 diabetes is that these individuals are at a markedly higher risk for premature cardiovascular events compared to the general population despite lack of traditional risk factors; indeed, type 1 diabetic individuals frequently have normal plasma cholesterol levels [1]. Current American Diabetes Association guidelines recommend statin therapy for people with diabetes (both type 1 and type 2) over the age of 40 regardless of baseline lipid levels for the purpose of CVD prevention [15]. Individuals with insulin resistance and type 2 diabetes have been observed to have lower absorption and higher synthesis of cholesterol [16]–[18]. By contrast, type 1 diabetic individuals have been proposed to have reduced synthesis and elevated absorption of cholesterol [16]–[18]. This discrepancy in relative cholesterol synthesis begs the question of whether statin therapy is warranted in type 1 diabetic individuals, given that it has been argued that while the benefits of statin therapy for CVD prevention is well established in type 2 diabetes, there is a lack of sufficient evidence that the same is true for type 1 diabetes as many large clinical trials have been underpowered to answer this question [19]. The utility of statins for cholesterol reduction in type 1 diabetes is particularly relevant given that differences in cholesterol synthesis may predict response to cholesterol-lowering treatment [20]. Therefore, determination of cholesterol synthesis in this group is clinically relevant because it may lend insight into appropriate treatment modalities. Previous studies have been conducted using sterol markers, which can be used to estimate cholesterol synthesis and absorption but are qualitative in nature [21]. The deuterium incorporation method is useful for studying in vivo synthetic pathways [22] and can be used to estimate FA and cholesterol synthesis simultaneously [23]. In addition, the type 1 diabetic patients in previous studies tended to be younger, and therefore investigation of lipid synthesis in subjects with long-standing diabetes is warranted.

The objective of this study was to objectively measure DNL and cholesterol synthesis in individuals with long-standing type 1 diabetes who are otherwise free of diabetic complications, under simulated free-living conditions and using the deuterium incorporation method which is ideally suited to studies of lipid synthesis in short (<24 h) duration. It was hypothesized that, given the labile glycemic control in this population, DNL would be elevated compared to healthy non-diabetic individuals. In addition, it was hypothesized that the objectively measured rate of cholesterol synthesis would be reduced in individuals with type 1 diabetes compared to healthy non-diabetic subjects, based on previously published literature using sterol markers.

## Methods

### Subject Recruitment

Individuals with long-standing type 1 diabetes (n = 9) (duration of diabetes ranged 20–45 years; negative C-peptide (0.01±0.04 nmol/L); HbA1c 8.9±1.5%) were recruited from waiting lists for the University of Alberta Clinical Islet Transplant program. Islet transplant subjects have long-standing and labile diabetes characterized by severe recurrent hypoglycemia and hypoglycemic unawareness, but are free of unstable coronary artery disease, proliferative retinopathy and severe proteinuria [24], [25]. The Clinical Islet Transplant program in Edmonton, AB is the premier site for islet transplants in Canada - patients travel from across the country and spend only a few days in Edmonton. Therefore, control of assessments, pre-testing preparations, and sample size were restricted due to other appointments part of routine pre-transplant care provided by the Islet Transplant program.

Use of statin medication (78% of subjects) is common in diabetes for prophylaxis of vascular disease [15] and was stopped one week before testing. Cholesterol synthesis rebounds as early as 3 days [26] with complete wash out by 7 days of statin cessation [27], [28]; therefore a one-week washout period was deemed sufficient for analyzing short-term hepatic metabolism. As is common in this population [29], subjects were taking anti-hypertensive medication (100%) as well as 1-thyroxine (44%) in order to bring thyroid hormone levels to normal. Use of these medications was long-standing, and not modified for the testing period.

Healthy non-diabetic subjects (control; n = 9) were matched for ±10% of age and BMI to the diabetic subjects. Control subjects were not taking any medications, were non-smokers, normotensive, and normolipidemic, and had no diagnosed or family history of premature CHD or other metabolic disorders.

### Ethics Statement

Written informed consent was obtained from all subjects and this study was approved by the University of Alberta's Health Research Ethics Board.

### Study Protocol

**Figure 1:**
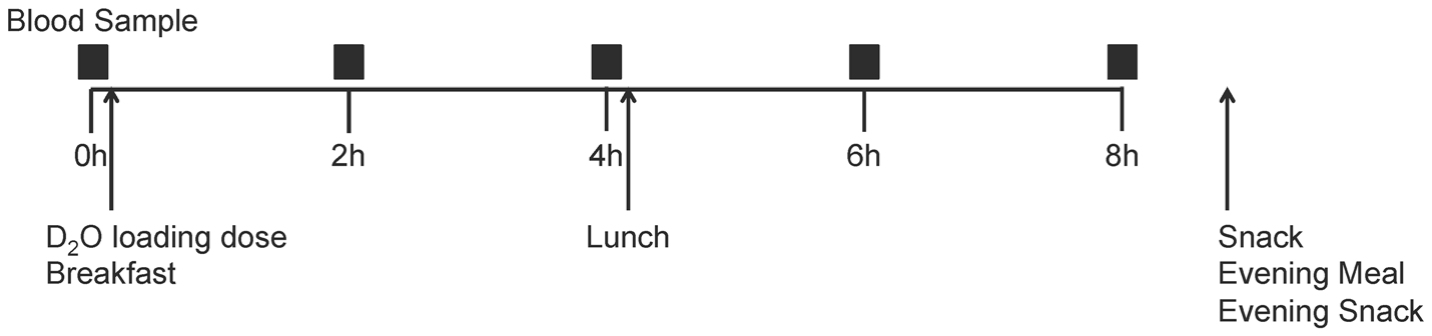
Schematic of the testing day design. Blood samples were taken at fasting (0 h) and every 2 h for 8 h after the D_2_O administration and breakfast completion. A lunch meal was provided after the 4 h sample, and take-away foods were provided for the subject to consume at home during the evening, before returning the following morning for a fasting 24 h blood sample.

On testing days (described in **Figure 1**), subjects arrived at the Human Nutrition Research Unit after an overnight fast. Blood samples were drawn and an IV catheter inserted for subsequent blood draws. Subjects consumed a loading dose of deuterium-labeled water (D_2_O; described below) following by breakfast. Postprandial timing initiated upon completion, and blood samples were drawn every 2 hours for 8 hours following breakfast. Lunch was provided after the 4 h sample, and take-home meals (described below) were provided for the subject to consume at home after the 8 h sample. Subjects were instructed to fast after 8 PM and returned the following morning for a 24 h blood sample.

Between the 2 h and 4 h samples, subjects' height and weight was measured using electronic scales, total body fat was measured by dual x-ray absorptiometry (GE Lunar Prodigy High Speed Digital Fan Beam X-Ray-Based Densitometer; GE Healthcare; Waukesha, WI) or BodPod (Life Measurement Inc.; Concord, CA), and blood pressure and waist circumference were measured by standard methods.

Subjects with diabetes were maintained on basal bolus insulin regimens comprised of both long- and short-acting insulins (glargine and lispro). Subjects with diabetes self-administered insulin injections at doses they themselves deemed to be appropriate to meet the labile nature of diabetes in this group, which was just before meals were consumed.

### Isotope Administration

For measurement of fatty acid and cholesterol synthesis, subjects were given a loading dose of deuterium-labeled water (D_2_O, D-175; 99.9 atom%; 1.0 g/kg body water estimated at 60% of body weight; D_2_O obtained from CDN Isotopes, Pointe-Claire, QC) to ingest before breakfast was consumed. The same dose of D_2_O (1.0 g/kg body water) was given in 1.5 L of water for subjects to consume throughout the day to maintain plasma deuterium levels. D_2_O was filtered through a 0.22 micron, 25 mm Cameo 25ES Cellulosic sterile and pyrogenic filter (GE Water & Process Technologies, Fisher Scientific; Ottawa, ON).

### Meal Determination and Feeding

Prior to testing day, subjects completed 3-day food record to estimate usual dietary intake. Food scales were provided to assist completion of food records, which were analyzed using Food Processor (ESHA Research, Versions 10.0–10.4; Salem, OR).

Meals (3 meals and 2 snacks) were provided in sequential fashion to standardize macronutrient consumption (20% of calories from protein, 45% carbohydrates, and 35% fat), elicit typical postprandial responses, and simulate free-living conditions. Meals consisted of whole foods and were provided at set times (nutrient breakdown of meals provided in **Table S1 in File S1**). Breakfast was given after the fasting blood sample was drawn and D_2_O administered, and included whole-wheat toast with margarine, scrambled eggs, fruit, and 1% milk. Lunch was provided after the 4 h blood sample had been drawn, and was comprised of pasta with chicken and vegetables in a marinara sauce, and 1% milk. A snack was provided after the 8 h blood sample and included raw vegetables with a low-fat dressing. An evening meal (sandwich containing whole-wheat bread, margarine, mustard, low-fat turkey, low-fat cheese, and tomato; and fruit) and a snack (hummus and whole-wheat pita bread) were provided to participants to consume at home. Non-caloric and non-caffeinated beverages with no added sugar or dairy were permitted throughout the testing period. Food quantity was scaled for each patient to mimic normal total caloric intake, estimated from the 3-day food records.

### Laboratory Methods

Blood was drawn into SST or K_2_EDTA Vacutainer tubes and centrifuged at 4°C for 10 min at 3000 RPM in a Jouan CR4.22 centrifuge and Jouan M4.4 rotor. Plasma and serum was stored at −80°C for analysis of plasma glucose, insulin, and lipids. Total cholesterol (TC) and HDL-c, TG, and glucose was determined by SYNCHRON-LX System using standard kits. LDL-c was calculated by Friedewald equation. Plasma C-peptide and HbA1c values were obtained from the Clinical Islet Transplant program.

### Lipid Analysis

Lipoproteins were obtained from 2 mL plasma and separated within 24 h of collection by sequential non-equilibrium density-gradient ultracentrifugation [2]. Briefly, plasma was layered with a 0.196 molal NaCl solution and ultracentrifugated twice using a Beckman Optima Centrifuge with MLS 50 rotor (25–30 min at 25,000 RPM and 20°C) to remove the chylomicron fraction. VLDL was extracted using 0.196 molal NaCl solution and centrifuged using a TL-100 centrifuge and Beckman TLA 100.2 100 K rotor (3 hours at 100,000 RPM at 20°C), and stored at −80°C. Lipids including TG, free cholesterol (FC), and cholesteryl ester (CE) were extracted and isolated from plasma and VLDL using a modified Folch procedure and thin layer chromatography (TLC) [2]. TG samples were saponified and methylated to FA-methyl esters for determination of FA composition using internal (15∶0) and external (GLC 461; Nu-chek Prep Inc.; Elysian, MN) standards to identify and quantify major FA (14–18 carbons in length and 20:4n6, 20:5n3, and 22:6n3). VLDL-TG FA methyl esters were analyzed on a Varian 3900 GC/MS (Varian Inc.; Palo Alto, CA) with a 30 m 0.25 mm BP20 column, and composition is expressed as % (wt/wt) total VLDL-TG FA.

### Lipogenesis and Cholesterol Synthesis

To isolate plasma water (PW) for deuterium enrichment analysis, plasma was filtered in 10 K polyethersulfone membrane centrifugal filters (VWR International; Mississauga, ON) for 1 h at 14,000 RPM on a Jouan A-14 centrifuge. Deuterium enrichment was measured using a High Temperature Conversion-Elemental Analyzer (Finnigan TC/EA) coupled to the Delta V Plus (oven temperature 90°C; pyrolysis reactor temperature 1400°C; helium flow 2.0 mL/min). Deuterium enrichment (δ‰) is expressed relative to VSMOW and in house standards of known enrichment [2], [30]. Samples were analyzed at each time point and injected 4–6 times for consistency, with the last 3 values averaged for calculations.

FA were isolated from VLDL-TG to describe hepatic de novo fatty acid synthesis (DNFA; %). Deuterium enrichment of VLDL-TG was analyzed using a Delta V Plus IRMS with Trace GC Ultra and Triplus Autosampler (Thermo Scientific; Mississauga, ON) with a 30 m BP20 column with 0.22 mm ID. Oven temperature was 90°C and ramped to 230°C, helium flow was 1.0 mL/min, injector temperature 240°C, and D/H reactor temperature 1420°C. Samples were analyzed in duplicate at each timepoint to represent fasting and postprandial synthesis, and FA areas and enrichment averaged for calculations.

FC and CE were isolated from plasma to describe whole-body cholesterol synthesis, and FC isolated from VLDL to more specifically describe hepatic cholesterol synthesis. Plasma FC and CE were measured at 0, 4, 8, and 24 h to capture synthesis during the morning (0–4 h), afternoon (4–8 h), and full 24 h, whereas VLDL-FC was measured at 24 h only. CE samples underwent further saponification and TLC to obtain the FC portion from these samples, which was kept separate for isotope analysis. FC and CE were run separately to determine fractional synthesis rate (FSR; % pool/day) for each. Both FC and CE samples were derivatized using 150 µL acetic anhydride and 40 µL pyridine [22], [31]. Samples were analyzed using a Delta V Plus with Trace GC Ultra using a 30 m 0.25 mm DB-5 column (Agilent) with 0.25 µm thickness. Oven temperature was 140°C, and ramped to 310°C, and then 320°C for post-run. Sample injection size was 1.0 µL, helium flow 1.3 mL/min, and injector temperature 280°C. Samples were analyzed in duplicate, and enrichment averaged for calculations.

### Calculations

DNFA for each major FA (14∶0, 16∶0, 16∶1, 18∶0, and 18∶1) was calculated [2], [32]:
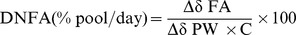
where Δδ FA ‰ and Δδ PW ‰ represent change in FA or PW enrichment from baseline (pre-dosing of ^2^H_2_O, time 0) to postprandial (post-dosing) samples. C is a constant calculated for each individual FA based on incorporation of 0.87 ^3^H per carbon of FA [33] and the ratio of carbon to hydrogen atoms in each FA as follows [2], [34]:
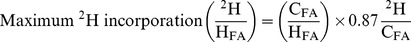
where C_FA_ is the number of carbons in the FA (e.g. for palmitate, C = 16) and H_FA_ is the number of potential hydrogens to be labeled in the FA (hydrogens associated with carbons; for palmitate H = 31). The calculated constants were 0.451 for 14∶0 (myristic acid), 0.449 for 16∶0 (palmitic acid), 0.480 for 16∶1 (palmitoleic acid), 0.447 for 18∶0 (stearate), and 0.475 for 18∶1 (oleic acid). Total DNFA was calculated by multiplying the calculated DNFAr% for each FA by the average area proportion (%) of each FA. The total overall de novo synthesis rate is calculated in this way to account for the fact that some FA (e.g. 14∶0) are more highly synthesized than others (e.g. 18∶0) but make up a smaller proportion of the total FA composition, which would bring the total overall FA synthesis rate lower.

Absolute rate of DNFA_-Abs_ (mg) was calculated [2]:
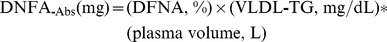
where VLDL-TG (mg/L) is estimated from the FA composition and quantity measured by GC, and plasma volume (L) is estimated for men (45 mL/kg) or women (37.5 mL/kg) [35].

Fractional synthesis rate (FSR; % pool/day) of FC, VLDL-FC and CE was calculated [36]:
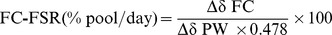
where Δδ FC ‰ represents change in deuterium enrichment in FC or CE, Δδ PW ‰ change in plasma water compared to baseline enrichment, and 0.478 represents maximal ratio of deuterium to hydrogen in newly synthesized cholesterol [36].

Absolute synthesis rate (ASR) of FC (g/day) was calculated [36]:
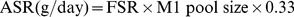
where M1 pool size reflects the rapidly exchangeable cholesterol pool and the constant 0.33 assumes the proportion of FC in the total cholesterol pool. The M1 pool size is calculated:
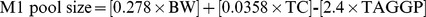
where BW is body weight (kg), TC is plasma total cholesterol (mmol/L), and TAGGP is a constant factor representing plasma TG level and denoted 1 (plasma TG<2.267 mmol/L), 2 (plasma TG 2.267–3.401 mmol/L), or 3 (plasma TG>3.401 mmol/L) [37].

### Statistical Analysis

Statistical analysis was performed using GraphPad Prism (V. 5.0c; GraphPad Software Inc.; La Jolla, CA). Groups were compared by Mann-Whitney non-parametric t-tests, or repeated measures ANOVA for postprandial data (P<0.05). Data presented as mean ± SEM.

## Results

**Figure 2:**
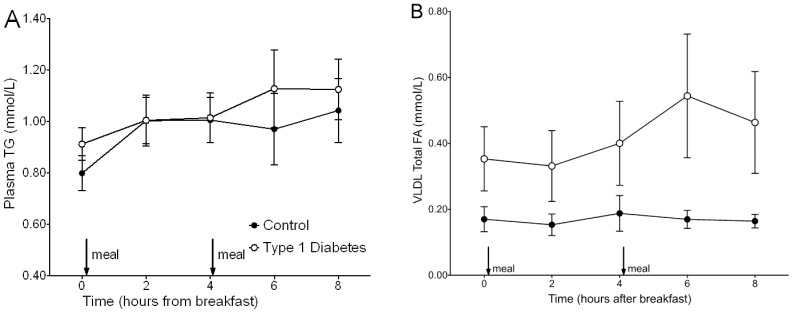
Postprandial levels of plasma- and VLDL-TG in control and type 1 diabetic subjects. Plasma-TG level (**A**) was higher in diabetic compared to control subjects at 6 and 8 h but not significantly different, whereas VLDL-TG concentration (**B**) and iAUC was greater in diabetic subjects (P<0.05), indicating postprandial lipemia. Arrows and “meal” on the figure indicate when a meal was fed at breakfast (time 0) and lunch (time 4 h). Black circles = control subjects; white circles = type 1 diabetic subjects; data presented as mean ± SEM.

There was no significant difference between groups in age, anthropometric, or most biochemical and dietary indices (**Table 1**), indicating successful matching of diabetic and control subjects. As expected, fasting plasma glucose was significantly higher in diabetic subjects and dietary fat intake was significantly higher while dietary sugar intake lower in the diabetic subjects. There was no significant difference between groups in LDL-c concentration, but previous data suggests that LDL-c levels may not return to baseline within 1 week of statin cessation [26], [27]. An analysis of total and LDL-cholesterol levels from patient histories when they were actively taking statins found both TC and LDL-c to be significantly higher at time of testing (i.e. after a week washout) (historic plasma TC during active statin treatment 3.73±0.11 mmol/L vs. 4.86±0.25 mmol/L after 1 week statin washout, P = 0.01; historic statin-treated LDL-c concentration 1.62±0.10 mmol/L vs. 2.85±0.31 mmol/L after statin-washout; P = 0.01). These data indicate there was at least a partial rebound of plasma cholesterol levels in the diabetic subjects. Fasting plasma TG levels were not different between groups, but tended to be higher at 6 h and 8 h during the postprandial period in diabetic subjects (**Figure 2A**) (P = 0.31). Similarly, fasting VLDL-TG concentration was not significantly different between diabetic (0.35±0.10 mmol/L) and control (0.17±0.04 mmol/L) (P = 0.29) subjects but postprandial VLDL-TG concentration (**Figure 2B**) and incremental area under the curve (iAUC) was significantly greater in diabetic compared to control subjects (p<0.05), suggesting delayed clearance of postprandial lipemia.

**Table 1:** Anthropometric and biochemical characteristics of control and diabetic subjects.

	Control	Type 1 Diabetes	P-value
*Anthropometric*			
Males∶Females	3∶6	4∶5	1.00
Age (years)	54±11	53±10	0.91
BMI (kg/m^2^)	25±2	26±4	0.65
Waist circumference (cm)	86±13	93±11	0.45
Body fat (%) - Males	26+7	21+9	0.40
Body fat (%) - Females	36+7	40+3	0.35
Systolic blood pressure (mmHg)	119±20	138±25	0.13
Diastolic blood pressure (mmHg)	74±19	88±14	0.21
*Biochemical Plasma Levels*			
Total cholesterol (mmol/L)	5.1±0.4	4.8±0.7	0.71
LDL-cholesterol (mmol/L)	3.1±0.5	2.9±0.5	0.62
HDL-cholesterol (mmol/L)	1.5±0.3	1.5±0.3	0.47
Triglyceride (mmol/L)	0.80±0.20	0.91±0.2	0.20
Glucose (mmol/L)	5.0±0.4	7.1±3.4	0.052
Insulin (mU/L)	5.7±4.4	3.4±3.6	0.20
*Dietary Intake* *			
Total kcal	1607±379	1756±638	0.84
Carbohydrate (%)	55±9	44±8	**0.02**
Protein (%)	19±4	21±4	0.38
Fat (%)	24±6	36±10	**0.02**
Saturated fat (%)	7.6±2.5	11.4±4.9	0.08
Monounsaturated fat (%)	7.6±2.7	11.1±6.6	0.25
Polyunsaturated fat (%)	4.4±1.8	5.6±3.0	0.53
Cholesterol (mg)	201±132	220±195	0.78
Fiber (g)	22±13	20±9	0.71
Sugars (g)	90±20	48±24	**<0.01**

* Dietary intake estimated from 3-day food records; dietary carbohydrate, protein, and fat are described as percent total energy intake. Data reported mean ± SD.

### De Novo Lipogenesis

**Figure 3:**
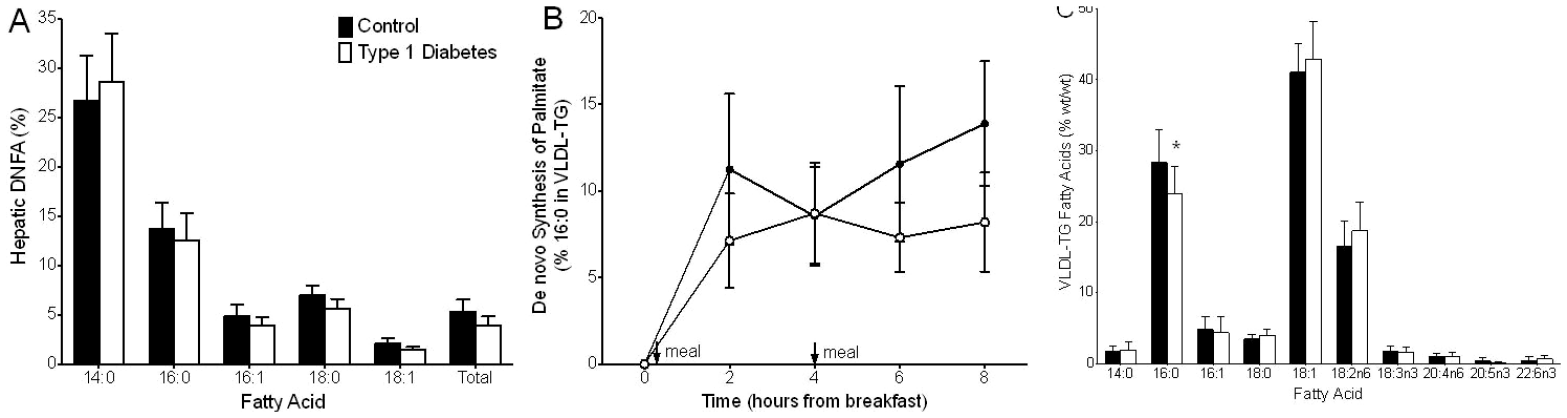
Fasting hepatic de novo fatty acid synthesis (DNFA) of total and individual fatty acids, postprandial synthesis of palmitate, and VLDL-TG fatty acid composition in control and type 1 diabetic subjects. *Panel A* - Fasting (24 h) total hepatic FA synthesis was similar between control and diabetic subjects (5.30±1.22% vs. 3.91±0.90%, respectively; P = 0.41), and was similar for individual FA including myristic acid (14∶0; 26.66±4.57% vs. 28.60±4.90%, P = 0.76), palmitic acid (16∶0; 13.71±2.64% vs. 12.52±2.75%, P = 0.65), palmitoleic acid (16∶1; 4.80±1.22% vs. 3.86±0.91%, P = 0.65), stearic acid (18∶0; 6.96±0.97% vs. 5.55±1.04%, P = 0.29), and oleic acid (18∶1; 2.10±0.51% vs. 1.45±0.28%, P = 0.21). *Panel B* – Similar to fasting, postprandial synthesis of palmitate, the major product of de novo lipogenesis, was not significantly different in diabetic compared to control subjects (P = 0.38). *Panel C* – Fatty acid composition of VLDL-TG was not different between groups except for 16∶0, which was significantly lower in diabetic subjects compared to controls (P = 0.015). Arrows and “meal” on Panel B indicate when a meal was fed at breakfast (time 0) and lunch (time 4 h). Black bars or circles, control non-diabetic subjects; white bars or circles, type 1 diabetic subjects; data presented as mean ± SEM.

Fasting hepatic synthesis of total and individual FA was not significantly different between groups (**Figure 3A**), nor was postprandial synthesis of palmitate (16∶0) (P = 0.38; **Figure 3B**). Similarly, absolute amount of newly synthesized total FA was not different between control and diabetic groups (DNFA_-Abs_; 22.25±5.09 mg vs. 51.75±27.89 mg, respectively; P = 0.94) nor was absolute amount of newly synthesized 16∶0 (13.65±3.13 mg vs. 36.25±20.15 mg; P = 0.80). Palmitate (16∶0) is shown as a representative FA for postprandial synthesis (**Figure 3B**) because it is the primary product of lipogenesis; individual rates of synthesis of palmitate are provided in **Figure S1 in File S1**. As can be observed in *Figure 2A*, myristic acid (14∶0) has a higher relative synthesis (i.e. a greater proportion of the total 14∶0 in VLDL-TG arises from de novo synthesis), but 14∶0 is a small contributor to overall VLDL-TG fatty acid composition, as can be appreciated in *Figure 2C*. This finding underscores the fact that absolute quantity of de novo synthesized FA is determined by both synthesis rate and TG concentration. DNFA% represents the *proportion* of newly synthesized FA in VLDL-TG, and reflects increased synthesis or contribution from this pool as compared to other FA sources available in the liver (e.g. NEFA, previously stored TG, and newly received dietary TG). VLDL-FA composition (% total TG-FA) (**Figure 3C**) was not different between groups except for 16∶0, which was lower in diabetic compared to control subjects (P = 0.02). De novo synthesis of individual FA was not correlated with the corresponding proportion contributing to VLDL-FA composition (P>0.05 for all FA). The ratio of 16∶0 to 18:2n6 has been proposed as a marker of lipogenesis. This ratio was not significantly different between groups (P = 0.13) but was marginally correlated with synthesis of 16∶0 (DFNA %) in control subjects (r = 0.62, P = 0.086) though not in the diabetic subjects (P = 0.84). Palmitate (as proportion of total VLDL-TG fatty acids) was positively correlated with saturated fat intake in control subjects (r = 0.67, P = 0.059) subjects, while it was negatively correlated with carbohydrate intake in diabetic subjects (r = −0.76; P = 0.037).

### Cholesterol Synthesis

**Figure 4:**
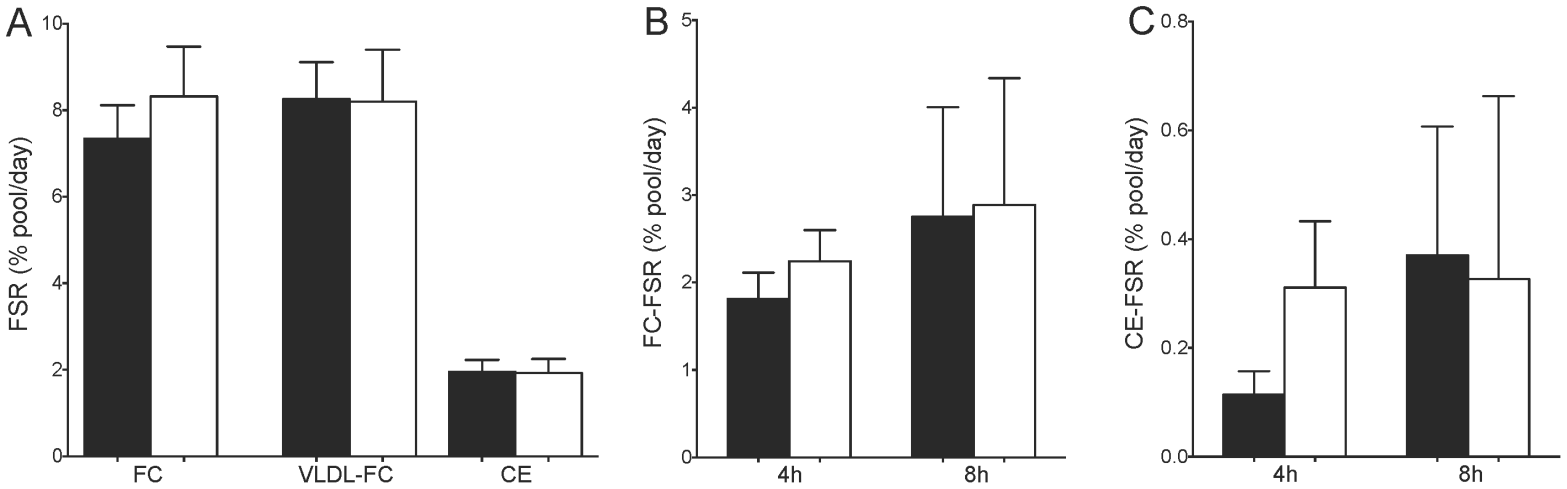
Synthesis of cholesterol determined from whole-body and hepatic free cholesterol (FC) and cholesteryl ester (CE) fractions at fasting and postprandial timepoints. Fasting FC-FSR was 7.3±0.8% in Control subjects compared to 8.3±1.2% in diabetic subjects (P = 0.65), VLDL-FC-FSR was 8.1±0.8% vs. 8.2±2.0% (P = 0.72), and CE-FSR was 2.0±0.3% vs. 1.9±0.3% (P = 0.93) (**A**). Postprandial FC-FSR (**B**) at 4 h (1.8±0.9% vs. 2.2±1.1%, P = 0.36) and 8 h (2.8±1.3% vs. 2.9±1.5%, P = 0.65) was similar between groups, as was CE-FSR (**C**) at 4 h (0.11±0.12% vs. 0.31±0.37%, P = 0.37) and 8 h (0.37±0.24% vs. 0.33±0.34%, P = 0.71). Black bars, control non-diabetic subjects; white bars, type 1 diabetic subjects; data presented as mean ± SEM.

Whole-body and hepatic synthesis of FC and CE was not significantly different between diabetic and control subjects (P>0.05 all) at fasting or postprandial timepoints (**Figure 4** **A–C**; individual rates of synthesis are provided in **Figure S1 in File S1**). Similarly, fasting FC-ASR was not different between diabetic (0.49±0.1 g/day) and control subjects (0.41±0.05 g/day) (P = 0.72). There was no difference in cholesterol synthesis in type 1 diabetic subjects who were taking (n = 4; FC-FSR 8.4±2.0%) and not taking 1-thyroxine (n = 5; FC-FSR 8.3±1.6%; P = 0.99). FC-FSR was negatively correlated with plasma TC (r = −0.81, P = 0.02) and LDL-c (r = −0.83, P = 0.02) in Control subjects while VLDL-FC-FSR should a trend towards correlating with LDL-c (r = −0.63, P = 0.076), suggesting greater cholesterol synthesis correlated with lower plasma cholesterol levels. In contrast, neither FC-FSR or VLDL-FC-FSR was correlated with plasma cholesterol levels in the diabetic subjects. FC-FSR was negatively correlated with dietary intake of cholesterol in diabetic (r = −0.75, P = 0.03) but not control subjects, suggesting lower cholesterol synthesis is associated with greater cholesterol intake in these subjects. Hepatic FC-FSR was not correlated with dietary cholesterol intake in either group. FC-FSR was also negatively correlated with saturated fat intake (as % of energy) in the diabetic subjects (r = −0.73, P = 0.03) but not in controls.

## Discussion

The current work provides evidence that lipogenesis and cholesterol synthesis are comparable in individuals with long-standing type 1 diabetes compared to age-, sex-, and BMI-matched controls. The data indicates that lipogenesis is not elevated in type 1 diabetes which is in contrast to other populations that experience impaired glycemic control such as type 2 diabetes and NAFLD [3], [4]. As such, lipogenesis likely does not contribute to the incidence of fatty liver that has been observed in these individuals [5]. Similarly, cholesterol synthesis in the present type 1 diabetic subjects was found to be comparable to that in non-diabetic controls, in contrast to previous studies reporting reduced cholesterol synthesis [16]–[18]. These findings are also in contrast to type 2 diabetic individuals, who are reported to have elevated cholesterol synthesis, and suggests that the routine prescription of statin medication to all diabetic individuals [15] should be re-evaluated for type 1 diabetic individuals.

### De Novo Lipogenesis

To the authors' knowledge this is the first investigation of hepatic synthesis of total and individual FA in humans with type 1 diabetes. The finding of similar rates of lipogenesis between diabetic and non-diabetic individuals in the present study is in contrast to the rates of DNL in type 2 diabetic subjects [2], despite a similar labeling procedure and timeframe. This discrepancy in magnitude of lipogenesis may be related to insulin resistance, because insulin resistance appears to have a greater influence on chronically elevating lipogenesis than does dietary intake. For example, individuals with insulin resistance, as occurring in type 2 diabetes [2] and NAFLD [3], appear to have high rates of lipogenesis in both the fasting and fed states, which does not display a circadian rhythm [3] or respond to changes in dietary intake as would be expected [2], [38]. Insulin resistance in type 1 diabetic individuals may be more common than previously appreciated [7], [8], but is difficult to assess in these individuals as it requires multi-stage clamp techniques and does not correlate with markers of glycemic control [7]. Despite the potential for insulin resistance to increase lipogenesis in type 1 diabetic individuals as it does in type 2 diabetes, lipogenesis may remain lower in type 1 diabetic patients due to the restriction of total energy intake and particularly dietary carbohydrate in these individuals. This dietary restriction could serve to limit lipogenesis even in the face of insulin resistance.

The low rate of lipogenesis observed in the present subjects (similar to non-diabetic subjects) is particularly interesting in this population given that it has been recently observed that a significant proportion of individuals with type 1 diabetes may have fatty liver [5]. In non-diabetic individuals with NAFLD, lipogenesis is elevated in subjects and contributes a substantial amount of FA to both VLDL- and intrahepatic-TG [3]. By comparison, other FA sources may contribute to hepatic lipid accrual in type 1 diabetes, including adipose-derived free FA (FFA) and dietary-derived TG. Plasma FFA concentrations were not measured in the present study, and is a limitation of this investigation, but has been previously shown to be elevated in type 1 diabetic individuals along with an impaired suppression of lipolysis with insulin administration [7], [8], [14]. Given the unpredictable nature of glycemic control and potential for insulin resistance (due to duration of diabetes and glycemic lability) in these individuals, it is likely that plasma FFA levels were elevated in these participants as well, contributing to FA flux to the liver and the elevation in postprandial plasma and VLDL TG levels noted here. In addition, dietary fat intake was greater in the present diabetic subjects compared to non-diabetic subjects. Therefore, primary sources of hepatic lipid in type 1 diabetic individuals are more likely to be derived from dietary fat and adipose lipolysis as opposed to lipogenesis. Ideally, a multiple stable isotope approach [3] to label various FA sources (lipogenesis, adipose, and dietary) could be utilized to determine the primary FA sources for VLDL- and intrahepatic-TG in type 1 diabetic subjects. Advice for individuals with NAFLD and type 2 diabetes for the purposes of reducing hepatic lipid accrual and lipogenesis would be to reduce overall caloric intake, with a particular focus on simple sugars [38], [39]; however, this counseling is already provided to type 1 diabetic individuals for glycemic control. Because lipogenesis does not appear to be elevated in the present cohort, clinical advice for managing lipid metabolism in type 1 diabetic patients could include modifying dietary intake to replace consumption of saturated fat with mono- and polyunsaturated fat which would be expected to have beneficial effects on insulin resistance and modify plasma, hepatic, and adipose fatty acid profiles.

The greater intake of dietary fat compared to carbohydrate of the diabetic subjects could contribute to the low observed lipogenesis, as it is generally accepted that dietary fat suppresses while carbohydrate stimulates DNL. However, if this were the case it would be expected that the control subjects would have had greater rates of lipogenesis compared to the diabetic subjects, given the higher habitual intake of carbohydrate. The relationship between carbohydrate intake and lipogenesis is contingent on the overall energy intake of the diet as well as the composition. In healthy individuals, DNL is elevated when carbohydrate is consumed *in excess* of energy needs [40]–[42] or when carbohydrate provides a significantly high proportion of energy (e.g. ∼70–80% energy from CHO) [38], [41], [43], [44]. However, the elevated DNL observed during high-carbohydrate feeding can be augmented by substituting simple carbohydrate for more complex forms, such as starch and fiber [45]. DNL can also be stimulated in the acute setting by providing a high-carbohydrate test meal comprised of mostly simple sugars (e.g. 80–100% of energy as CHO) [46], [47]. By contrast, the test day meals used in the present study were similar in fat (as % energy) to the background diet of the diabetic subjects, but were greater than the background diet of the control subjects. It is possible that lipogenesis in the control subjects may have been reduced as a function of the test day feeding. While it cannot be excluded, this is unlikely given that food quantity was provided in accordance with habitual overall energy intake, carbohydrate was provided in both simple and complex forms, and the difference in fat intake was likely not enough to significantly depress lipogenesis in these healthy subjects. The limited time frame available for testing the diabetic subjects precluded standardization of food intake leading up to the testing period and is a limitation of this investigation. In future studies it would be advantageous to provide standardized meals to all subjects for a 3-day run-in period.

### Cholesterol Synthesis

The present study provides evidence that the rate of cholesterol synthesis in individuals with long-standing type 1 diabetes is comparable to non-diabetic subjects. The finding that cholesterol synthesis may be reduced or at least comparable to non-diabetic subjects is interesting given that the majority of individuals with type 1 diabetes are treated with statin medication aimed at reducing cholesterol synthesis [15]. Interestingly, statin therapy may also upregulate intestinal absorption and synthesis [48]. While we did not assess cholesterol absorption or the relationship between cholesterol absorption and synthesis, previous studies have suggested that type 1 diabetes have relatively reduced cholesterol synthesis but greater cholesterol absorption [16]–[18]. If hepatic cholesterol synthesis in type 1 diabetes is comparable to healthy individuals, and if cholesterol absorption (and potentially intestinal cholesterol synthesis) are actually increased, perhaps a different treatment option targeting intestinal cholesterol regulation, such as ezetimibe or plant sterols, should be utilized in these patients. Because cholesterol absorption inhibitors may reciprocally increase hepatic cholesterol synthesis [49], a combination of both statins and ezetimibe or plant sterols may prove to be most effective in this population. Indeed, a randomized double-blind parallel study of type 1 diabetic patients treated with statins found that the addition of plant sterols (contained in a food spread, providing 3 g/day plant sterols) further reduced serum cholesterol by 10%, LDL-c by 16%, and non-HDL-cholesterol by 15% with no effect on HDL-c [50]. Further, one of cholesterol-lowering mechanisms of statins is the upregulation of LDL-receptors as compensation for reduced hepatic cholesterol synthesis [51]. This feature of statins may be particularly important in type 1 diabetic individuals because evidence suggests there may be impaired lipoprotein clearance in these patients, supported here by the slightly elevated postprandial plasma TG levels at 6 and 8 h, and previous findings of elevated postprandial Apolipoprotein B48 levels in these patients, indicating impaired chylomicron clearance [52]. Postprandial lipemia has been suggested to be particularly atherogenic in diabetes due to finding of greater remnant particle binding capacity to arterial proteins, leading to potentially greater arterial cholesterol deposition [53]. Finally, given the poly-pharmacy and related financial burden already incurred by type 1 diabetic patients, a combination drug-dietary therapy, such as statins and plant sterols, may be ideal in this population [54]. Therefore, the present data suggests that pharmacological agents targeting cholesterol absorption, such as ezetimibe or plant sterols, may be more effective to reduce plasma cholesterol and reduce CVD risk in type 1 diabetic individuals than agents singularly targeting cholesterol synthesis, such as statins. The outcomes of current clinical trials testing this hypothesis may provide answers (ClinicalTrials.gov registrations NCT00477204 and NCT00879710).

In previous reports, cholesterol synthesis was lower in individuals with type 1 diabetes compared to both non-diabetic and type 2 diabetic subjects [16]–[18]. Discrepancies between the current report and previous investigations may be due to differences in methodology as well as patient selection. Previous reports used sterol markers to estimate cholesterol synthesis, which correlate with the deuterium method [55] but are limited to qualitative descriptions of cholesterol metabolism [21]. Sterol markers were not measured in the present study, which is a limitation of this investigation, but would be advantageous to include in future studies to provide additional indicators of synthesis as well as absorption. A further difference between the present study and previous investigations is that subjects in previous studies tended to be younger (age ∼30) and have better glycemic control (A1c ranged 6–8%). Finally, though not assessed in this study, it is becoming increasingly appreciated that subjects with type 1 diabetes may also be insulin resistant [7], a state which is associated with elevated cholesterol synthesis [16]–[18]. Therefore, differences in glycemic control may underlie differences in the present observations, suggesting cholesterol synthesis may increase with worsening glycemic control. However, further analysis by Gylling and colleagues suggested that improving A1c in diabetic subjects actually increased markers of cholesterol synthesis [56] and therefore this concept requires further investigation.

Despite the association of dietary saturated fat intake with plasma cholesterol levels, the effect of dietary fat on cholesterol synthesis in particular is more complex. In particular, there are few studies addressing how total dietary fat intake affects cholesterol synthesis. What has been shown is that significantly restricting total dietary fat intake (to 15% of calories) reduces cholesterol synthesis similar to that achieved by simple restriction of total energy intake (achieved through limiting primarily dietary carbohydrate) [57], whereas changing dietary fat composition appears to induce more apparent differences in cholesterogenesis. Specifically, cholesterol synthesis is higher following PUFA consumption, particularly for n-6 PUFA vs. n-3 PUFA, as compared to SFA and MUFA [58], [59]. It is not clear whether the greater total dietary fat intake of the diabetic subjects in the present investigation affected the observed rate of cholesterol synthesis; however, the relative proportions of saturated to monounsaturated and polyunsaturated fat were similar between groups and therefore are unlikely to have impacted the observed findings.

In contrast to dietary fat, dietary cholesterol tends to have a neutral effect on cholesterol synthesis; however, increasing cholesterol intake can modestly reduce cholesterogenesis [60]. An inverse relationship between plasma cholesterol levels and synthesis has been reported previously [61] and appears to be especially important in individuals who have lower rates of synthesis; these individuals tend to exhibit the largest increase in plasma cholesterol after a dietary cholesterol challenge which suggests a greater role for cholesterol absorption in these individuals. The present findings do not suggest reduced cholesterol synthesis compared to non-diabetic individuals, but synthesis was observed to be inversely related to dietary cholesterol intake in the type 1 diabetic subjects; this finding would support the potential for elevated cholesterol absorption.

### Limitations

The limitations of this study warrant discussion. As described previously, the effect of statins to reduce cholesterol synthesis is abolished by as early as 3 days after statins are ceased [26]; by comparison, LDL-cholesterol levels may not return to statin-naïve levels by 7 days of statin washout [26], [27]. Total and LDL-cholesterol levels from patient histories were significantly higher at time of testing, but it is not possible to determine if LDL-c levels would have increased further after a longer washout period. This may explain why plasma lipid levels were similar between the diabetic and control subjects. However, diabetic individuals frequently have cholesterol levels comparable to non-diabetic individuals [1]; therefore it is unlikely that LDL-c levels in our diabetic subjects have been grossly underestimated. Nonetheless, the effects of statins on cholesterol synthesis are washed out by 7 days, and the rates of cholesterol synthesis measured from whole plasma and isolated VLDL were similar between the type 1 diabetic and control subjects. Finally, the type 1 diabetic subjects in the present study had long-standing and labile diabetes; therefore the results herein may not extend to all individuals with type 1 diabetes, particularly those with good glycemic control. However, this group was chosen because of their poor glycemic control yet relative lack of complications (renal disease, advanced cardiovascular disease) that could otherwise complicate the measurements of lipid metabolism.

## Conclusions

The present work indicates that lipid synthesis is not significantly different in individuals with long-standing and labile type 1 diabetes compared to health non-diabetic adults. First, the data suggests lipogenesis is not elevated in type 1 diabetic individuals and likely is not a major contributor to circulating or stored triglyceride levels in these subjects, in contrast to other patient groups also at elevated risk of vascular disease, such as type 2 diabetes and NAFLD. Indeed, fatty acids arising from diet and stored adipose tissue may have a greater influence on hepatic metabolism in type 1 diabetes than in other populations where insulin resistance profoundly elevates lipogenesis as a significant source of fatty acids in the liver. Second, the finding of cholesterol synthesis and plasma cholesterol levels in type 1 diabetic individuals with poor glycemic control that are comparable to healthy non-diabetic individuals suggests that the routine use of statin medication for CVD reduction in this group should be evaluated within the context of other treatment options available which may instead modify cholesterol absorption, such as ezetimibe or plant sterols. In conclusion, the data indicates that metabolic risk in type 1 diabetic subjects may be beneficially managed by a combination of dietary and pharmaceutical interventions. Future research is required to delineate the possible interplay of lipogenesis, free fatty acid metabolism, insulin resistance and hepatic steatosis in this group, as well as the investigation of alternative cholesterol-lowering treatments in this group for prophylaxis of vascular disease.

## Supporting Information

File S1 **Supporting Table S1 and Figure S1.**
(DOCX)
