# Supplementary material for: De Novo Lipogenesis and Cholesterol Synthesis in Humans with Long-Standing Type 1 Diabetes Are Comparable to Non-Diabetic Individuals
Source: PLoS One. 2013 Dec 23;8(12):e82530. doi: 10.1371/journal.pone.0082530 (PMC3871159; doi:10.1371/journal.pone.0082530)
Supplement: File S1 — Supporting Table S1 and Figure S1. (DOCX) [file pone.0082530.s001.docx]

**“De novo lipogenesis and cholesterol synthesis in humans with long-standing type 1 diabetes are comparable to non-diabetic individuals”**

**Supplementary Information**

Contents:

**Table S1**: Nutritional information of meals provided during the intpatient testing period.

**Supplementary Figure S1**: Individual rates of synthesis of palmitate (**A**) as well as free cholesterol (FC) isolated from whole plasma or VLDL and cholesteryl ester (CE) (**B**).

Table S1: Nutritional information of meals provided during the study.

| **Meal** | **Total Energy (kcal)** | **Fat (%)** | **Carbohydrate (%)** | **Protein (%)** |
| --- | --- | --- | --- | --- |
| **Breakfast** | 384 ± 104 | 46 | 43 | 14 |
| **Lunch** | 362 ± 105 | 20 | 52 | 28 |
| **Afternoon Snack** | 123 ± 33 | 45 | 54 | 8 |
| **Evening Meal** | 417 ± 123 | 34 | 44 | 25 |
| **Evening Snack** | 411 ± 110 | 35 | 57 | 14 |
| **Daily Total** | 1697 ± 476 | 35 | 49 | 19 |

- There was no difference in gram quantity of total foods or any individual food provided between the control and type 1 diabetes groups therefore nutrient analysis was pooled and reported as the average across all subjects.
- The following food items were provided for each meal, as described in the text:

- Breakfast: whole-wheat toast with margarine, scrambled eggs, fruit, and 1% milk

- Lunch: pasta with chicken and vegetables in a marinara sauce, and 1% milk

- Afternoon snack: raw vegetables with a low-fat dressing

- Evening meal: sandwich containing whole-wheat bread, margarine, mustard, low-fat turkey, low-fat cheese, and tomato; and fruit

- Evening snack: hummus and whole-wheat pita bread

**Supplementary Figure S1**: Individual rates of synthesis of palmitate (**A**) as well as free cholesterol (FC) isolated from whole plasma or VLDL and cholesteryl ester (CE) (**B**).
